# Inter-paralog amino acid inversion events in large phylogenies of duplicated proteins

**DOI:** 10.1371/journal.pcbi.1010016

**Published:** 2022-04-04

**Authors:** Stefano Pascarelli, Paola Laurino

**Affiliations:** Protein Engineering and Evolution Unit, Okinawa Institute of Science and Technology Graduate University, Onna, Okinawa, Japan; University of Haifa, ISRAEL

## Abstract

Connecting protein sequence to function is becoming increasingly relevant since high-throughput sequencing studies accumulate large amounts of genomic data. In order to go beyond the existing database annotation, it is fundamental to understand the mechanisms underlying functional inheritance and divergence. If the homology relationship between proteins is known, can we determine whether the function diverged? In this work, we analyze different possibilities of protein sequence evolution after gene duplication and identify “inter-paralog inversions”, i.e., sites where the relationship between the ancestry and the functional signal is decoupled. The amino acids in these sites are masked from being recognized by other prediction tools. Still, they play a role in functional divergence and could indicate a shift in protein function. We develop a method to specifically recognize inter-paralog amino acid inversions in a phylogeny and test it on real and simulated datasets. In a dataset built from the Epidermal Growth Factor Receptor (EGFR) sequences found in 88 fish species, we identify 19 amino acid sites that went through inversion after gene duplication, mostly located at the ligand-binding extracellular domain. Our work uncovers an outcome of protein duplications with direct implications in protein functional annotation and sequence evolution. The developed method is optimized to work with large protein datasets and can be readily included in a targeted protein analysis pipeline.

## Author summary

Proteins are critical components of living systems because they facilitate most biological processes like protein synthesis, DNA replication, chemical catalysis, etc. Proteins are encoded in their genes. During evolution, genes accumulate mutations that get translated at the protein level. These mutations can be “neutral” if they do not affect the protein function immediately and directly; otherwise, mutations can be functional if they directly modify protein function. An event that provides an opportunity to study protein function is gene duplication namely, when two copies of a gene encoding the same protein appear. One copy of the protein often retains the same function while the other is free to diverge and specialize to a different function. This work sheds light on an alternative outcome of gene duplication that might be critical to discern between neutral and functional mutations. By looking at 88 fish genomes, we found proteins in which the evolution of their sequences does not follow the expected pattern of divergence after gene duplication. In this case, the protein sequence of a subgroup of species diverges in the copy expected to retain its function, while the sequence is retained in the expectedly divergent one. We called this event “inter-paralog amino acid inversion”. Our data shows that this “inversion” event is correlated to function, and its detection has to be considered for assigning protein functions correctly.

## Introduction

Proteins perform their function either through protein-protein interactions, protein-ligand interactions, or catalyzing chemical reactions. At the molecular level, amino acids of a protein interacting with a counterpart, namely a ligand (small molecule, protein, DNA/RNA, etc.) are herein defined as “functional residues”. The importance of predicting functional residues in a protein is evident as these residues can contribute to designing new functions, switching specificities, defining protein families and subfamilies, or identifying the occurrence of a functional innovation (e.g., a change of ligand specificity). Crystal structures in which the protein of interest was co-crystallized with its ligand, can readily identify functional residues. However, when the structure is not available, the identification of functional residues is not trivial. Previous attempts used the evolutionary information found in protein sequences and their homologs [1–3], approaches now facilitated by the global-scale genome sequencing effort driven by the development of high-throughput sequencing technologies. The prediction of functional residues by such methods is hampered by the presence of neutral substitutions, namely amino acid substitutions that are neither beneficial nor disadvantageous [4]. Non-synonymous neutral substitutions are on average 10 times more abundant than the advantageous ones [5]. While neutral substitutions are directly proportional to the time of divergence, a change in functional residues could be a signal of a functional shift that might occur independently of the divergence time. When the relationship between phylogeny and function is decoupled, neutral substitutions may mislead homology-based prediction methods, which are the most common way of functional prediction [6].

An event that often decouples the phylogenetic and functional signal is gene duplication. After gene duplication, two proteins follow a semi-independent evolution. For example, before diverging, the two duplicates may influence each other by gene conversion [7] or homomeric-heteromeric interactions [8], and they tend to diversify in the expression profile [9,10]. Gene duplication is prevalent in all domains of life [11], and often the duplicated proteins are reported to go through functional diversification [12]. For example, in an event termed “sub-functionalization”, a protein with multiple functions (e.g., cellular receptors binding multiple ligands) might split its functions between the two gene copies after duplication. Previously, McClintock *et al*. showed that, in zebrafish HOX genes, the subset of functions inherited by the duplicated copies is different between fish and mouse–a phenomenon named “function shuffling” [13]. In cases alike, the phylogenetic signal is misleading when used to predict the function of a “shuffled” orthologous protein. However, if the functional divergence is correctly identified, it allows to highlight the functional residues responsible for this transition, with reduced noise from the neutral variants. In this work, we address the identification of protein functional residues that are mutated during this type of functional rearrangement.

Functional residues responsible for a change of function within a protein family are usually called Specificity Determining Sites (SDS). SDS can be predicted by multiple methods [14]. SDS prediction methods use the Multiple Sequence Alignment (MSA) or the 3D structure of the protein of interest to calculate a score based on conservation [15–23], evolutionary rate [24–26], or 3D structure properties [27]. Most of these approaches require the user to provide the correct groupings of the homologous proteins. When this information is missing, the groupings are made according to the ortholog classification obtained by manual or automatic partitioning methods [28–31]. However, the SDS predictions in automatically partitioned orthologs showed a lower sensitivity [32], demonstrating that an incorrect grouping negatively influences the prediction. The grouping usually follows the ortholog conjecture, namely that orthologs are more conserved than paralogs [33,34]. When this is not true, the SDS prediction is hampered. Therefore, the power to predict functional residues is limited by our ability to track protein function on the phylogenetic tree when it is not vertically inherited by orthologs. In our work we address this problem by identifying a signal of functional transition that might prove to be useful when annotating orthologs.

EGFR (Epidermal Growth Factor Receptor) is a tyrosine-kinase receptor that activates multiple signaling pathways after binding one of the seven EGFR ligands [35]. EGFR is broadly expressed [36] and plays a crucial role in several aspects of organismal development and homeostasis like cellular growth, differentiation, metabolism, and motility [37]. In fish, two copies of EGFR were kept after the Teleost-Specific Genome Duplication (TSGD) event that occurred about 350 mya in the actinopterygian lineage [38,39]. Lorin *et al*. showed that both copies of EGFR might have been retained because they are involved in the complex process of skin pigmentation [40], a trait that is under selective pressure in most fish. Furthermore, the extracellular domain of fish EGFR, responsible for binding multiple ligands, likely went through sub-functionalization [41]. For these reasons, EGFR constitutes a perfect model to study uneven functional inheritance events.

In this work, we observe a scenario where the function of a protein is not linearly inherited across orthologs, and we identify the functional residues responsible for the shift of functions. Our goal is to develop an algorithm that highlights the signature of a putative inversion of function, as could be an inversion of amino acids between paralogs within the same species. First, we obtain a simple theoretical model that describes the likelihood of an inter-paralog inversion in comparison to other outcomes. Then, based on the model, we develop an algorithm that identifies inter-paralog inversions in a phylogeny, and we apply it in the context of fish EGFR duplication. Finally, we validate the results using statistical scores and simulated evolution. Our analysis shows a new way to investigate an important and understudied outcome of gene duplication.

## Results and discussions

### Theoretical model for the identification of inter-paralog amino acid inversions

**Fig 1:**
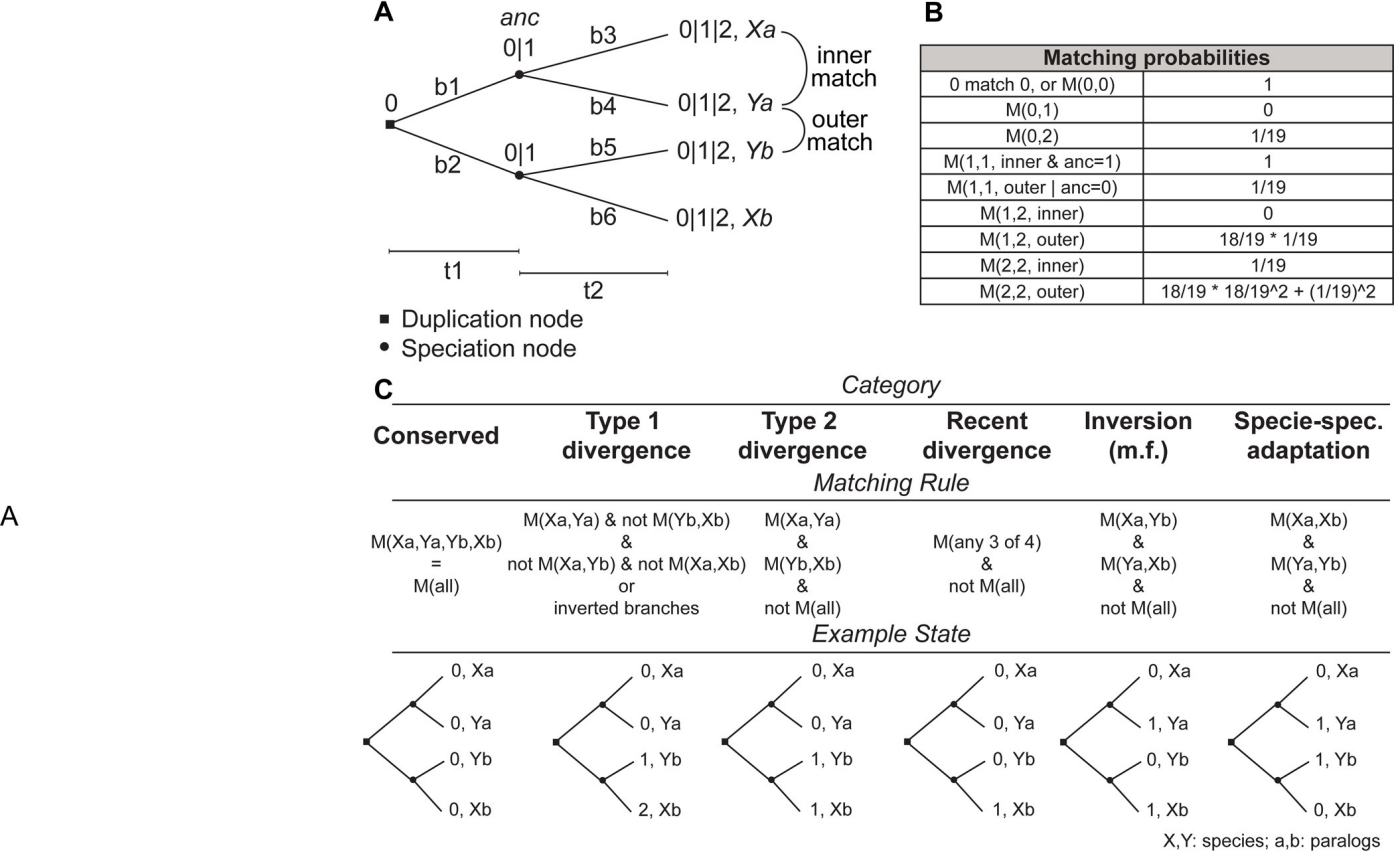
Theoretical model of the evolution of protein site after gene duplication. **(A)** The structure of the phylogenetic tree that the model is based on. The branch lengths t1 and t2 are used to determine the probability of a substitution on each branch b1 to b6. A leaf node can be found in states 0, 1, or 2 depending on the number of mutations in the preceding branches. An inner match is defined to be a match between orthologs (Xa to Ya, or Xb to Yb), while an outer match is any other match. The probability for a match between two states is given by the table in **(B)** and represents the underlying transition to any of the 20 amino acids. **(C)** Description of the categories. The categories represent a biologically interpretable situation, suggested by their name. Given a certain outcome configuration of states, it is possible to calculate the probability of observing a certain category by using the matching rule. The “Example State” section shows the leaf configuration that gives the highest probability of observing the category described.

First, we constructed a simplified model to describe the evolution of a site through a protein phylogeny after gene duplication and speciation. In the model, a tree branch (b1–b6) could be either substitution (1) or no-substitution (0) state, while a leaf node (Xa, Ya, Xb, Yb) has the possible states 0, 1, or 2 depending on the number of substitutions in the preceding branches (Fig 1A). A configuration of six branch states univocally leads to a configuration of four-leaf nodes. The model uses two branch length parameters, pre-speciation (t1) and post-speciation (t2), to calculate the probability of each of the 64 branch configurations. Using the model and a set of matching rules between the leaves (Fig 1B), we assessed the probability of seven categories/scenarios of configurations (Fig 1C): *Conserved*, all four leaf node states match; *Type 1 divergence*, leaf node states match in only one paralog group; *Type 2 divergence*, leaf node states match per paralog group but not per species; *Recent divergence*, all leaf node states match except for one; *Inversion*, leaf node states of opposite paralog group match but not per species; *Species-specific adaptation*, leaf node states match per species, but not per paralog group; and *Non-conserved*, collecting all events that do not fall in any of the other categories. We used the following formula to calculate the probability of each category:

$$P_{cat}=\sum_{s}^{N}P_{conf}*P_{\left(cat|conf\right)}$$

Where N are the 64 configurations of branch states, the probability *P_conf_* of the branch configuration is given by the model (S1 Fig), and the conditional probability for the category *P*_(*cat|conf*)_ is determined by the category’s matching rules.

**Fig 2:**
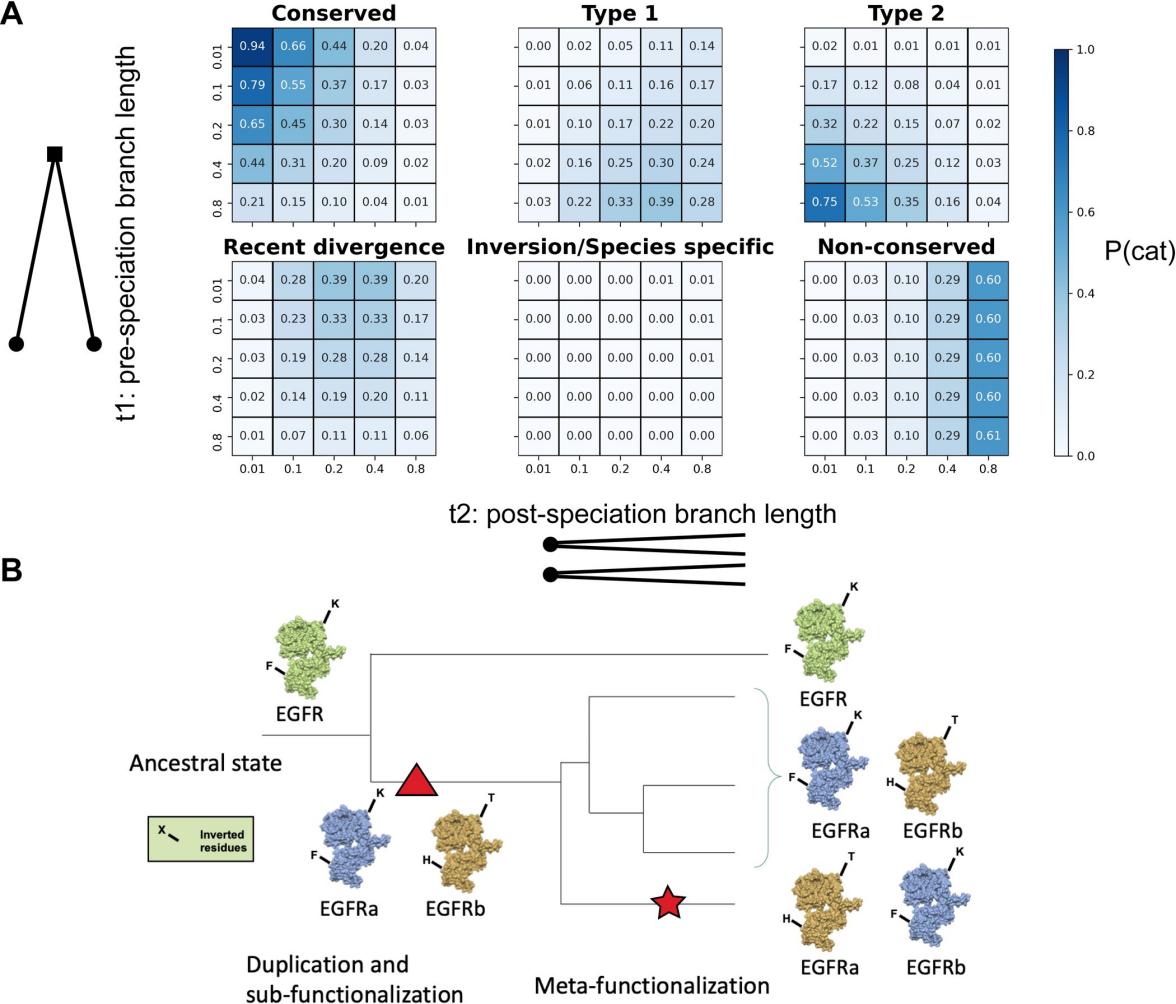
Theoretical model results. **(A)** The heatmaps show the category probabilities at different tree branch lengths as calculated using the theoretical model definitions. **(B)** Exemplification of “Meta-functionalization” (star), the putative driver of inter-paralog inversions in the phylogeny. A multifunctional protein (green) subdivides its functions (blue and yellow) between the two copies obtained after gene duplication (triangle). In a sub-group of species, the functional inheritance of the two copies is inverted. This event is revealed by the pattern of amino acids inversions compared to the majority of the other species.

Next, we tested the probability of each category at varying branch lengths (Fig 2A). As expected, short branch lengths lead to a high probability of conserved sites, and long branch lengths lead to non-conserved sites. When the pre-speciation branch length is the longest of the two, we observe a high probability of Type 1 and Type 2 divergence. Whereas, when the post-speciation branch length is the longest, we observe a high probability of recent divergence. Interestingly, for any branch length combination, the two categories of inversion and species-specific adaptation have less than a 1% chance of appearing. We do not expect to see inter-paralog inversions and specie-specific adaptations in real data because of their low probability of occurrence.

From the previous results, inter-paralog amino acid inversions should not be recurrent in a phylogeny. Thus, an unlikely high presence of these inversions may show that: 1) the involved sites are following a selective pressure directly related to the paralog function that, for a particular subgroup of species, is opposite and complementary to the other species; or 2) these sites have an indirect epistatic effect. Recurrent inversions could be the proxy of a functional rearrangement between the paralogs within a clade. A rearrangement of functions is facilitated in, but not limited to the Innovation Amplification Divergence (IAD) model of duplicates divergence [42], where paralogs partially retain their secondary functions. We describe this subgroup-specific functional inversion event as a possible outcome after the sub-functionalization of a duplicated gene and we suggest the name “meta-functionalization” from the Greek word “metathesis”, namely “put in a different order” (Fig 2B). This event is a special case of parallel evolution where the two paralogs concurrently converge to opposite fates in a sub-clade of the duplication tree.

### Algorithm for the identification of inter-paralog amino acid inversions

We developed an algorithm to identify the events of inter-paralog inversion in a protein phylogeny using a multiple sequence alignment (MSA) and a phylogenetic tree. The algorithm was implemented as a python package named DIRphy, for the Detection of Inverted Residues in a phylogeny. DIRphy splits the protein sequences of the MSA into four groups according to the organism and ortholog annotations, which can be either provided by the user or automatically done using a tree distance parameter. Based on the matching probabilities of the previous theoretical model, DIRphy calculates a score for each event of “Inversion” and “Species-specific adaptation”, representing its probability to occur (see methods for details). However, for this paper, we will mainly focus on the inversion events. The output of DIRphy is a list of positions above a defined threshold. When the organism grouping is manually selected, the script calculates both the observed and the expected probability of inversion between the specified groups in the given tree. Otherwise, when the organism grouping is not specified, the output table shows the observed probability of amino acid inversions given by the grouping that has the highest probability for that position. In the current version of DIRphy, only a binary paralog classification is allowed. DIRphy is released as an open-source project in Github: https://github.com/OISTpasca/protein-inversions

### Construction of a fish EGFR dataset and identification of inter-paralog amino acid inversions

We tested DIRphy in the phylogenetic tree of the Epidermal Growth Factor Receptor (EGFR) in the fish lineage. First, we filtered 88 fish genomes (taxon 41665) from the European Nucleotide Archive (ENA) [43] to obtain a dataset of 167 fish EGFR protein sequences. The dataset included all high-quality (N50 > 1Mb) teleost genomes plus one outgroup before the TSGD (spotted gar). From the phylogenetic analysis of this dataset (S2 Fig), three clear duplication events can be observed. The first, most ancient duplication coincided with the TSGD, around the time of the split with gars (~350 mya), and resulted in the separation of the two copies EGFRa and EGFRb. Two more copies were found in most salmonids, corresponding to the salmon-specific whole-genome duplication around ~80 mya [44], and one more copy in goldfish, possibly due to the carp-specific whole genome duplication about ~10 mya [45]. The longer branch lengths of EGFRb (Mann-Whitney test, p-val: 2.305e-11) indicate that EGFRb is evolving more rapidly than its counterpart. Furthermore, the EGFRb gene was more commonly lost. Out of 15 gene loss events, only one species lost EGFRa (*S grahami*), while 14 species lost EGFRb. We further confirmed the orthologous classification of EGFR duplication using the synteny analysis of ENSEMBL (S3 and S4 Figs and S2 Table).

**Fig 3:**
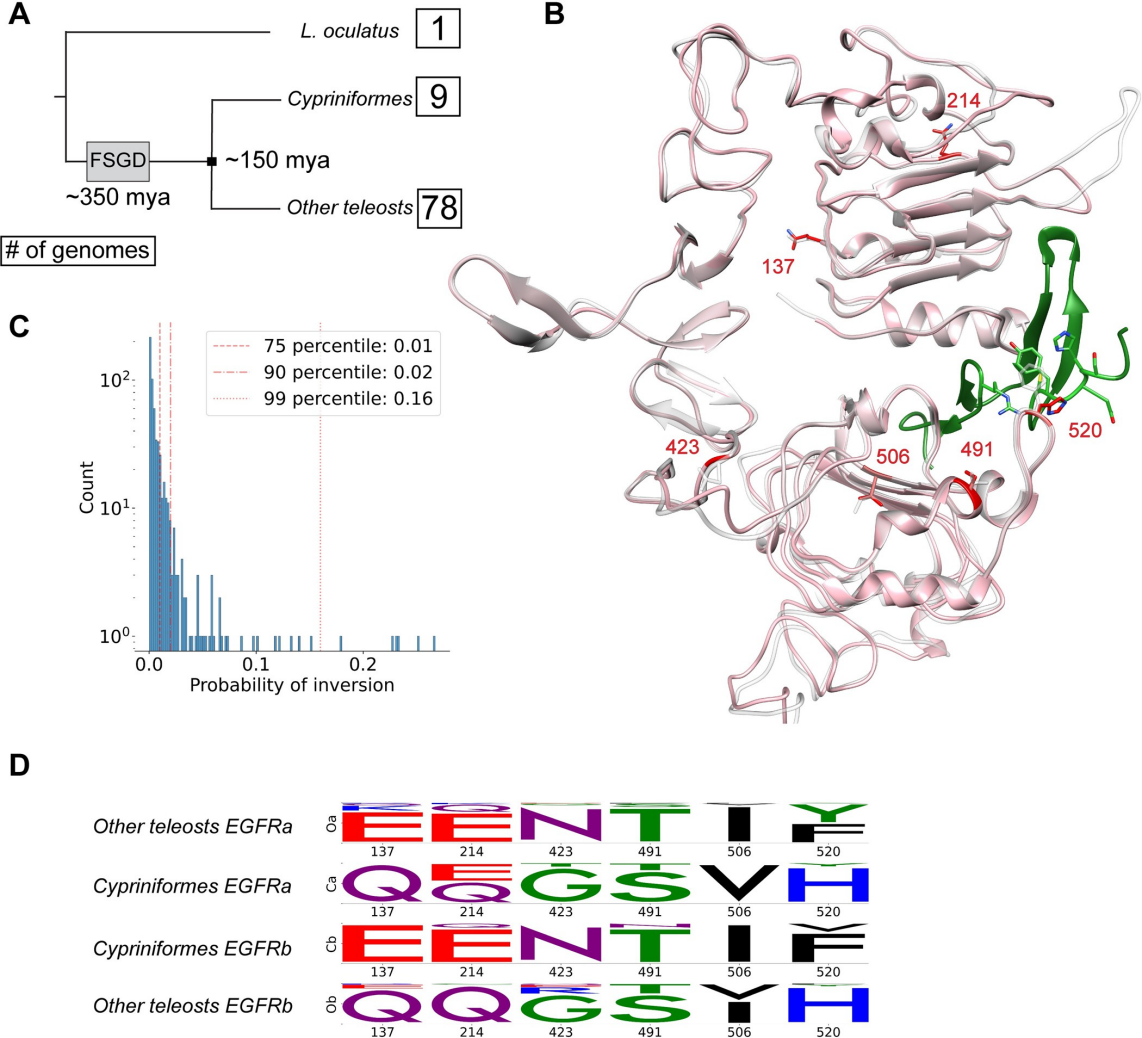
Inter-paralog inversions of amino acids in fish EGFR. **(A)** Schematic representation of the fish dataset phylogeny. The dates (mya: million years ago) indicate the time of the Fish Specific whole Genome Duplication (FSGD) and the separation of Cypriniformes fish to all other teleost fish. The number in the boxes represents how many genomes are in the dataset for that group. **(B)** 3D model superposition of *S*. *anshuensis* EGFRa (pink) and EGFRb (white), generated by homology using human EGFR as a template (1IVO) [48]. The inverted residues have been highlighted in red. The ligand EGF (green) was taken from the human model after superposing the receptors. **(C)** DIRphy score distribution. The inter-paralog inversion event probability score was calculated for each site in the MSA that has less than 60% gaps. The top 1% of sites were further characterized. **(D)** Logo representation of the four sub alignments (two species groups, two protein copies) in the inverted sites. The logo represents the normalized amino acid count per column and was obtained using the python package Logomaker [49].

Next, we tested the previously computed phylogeny and MSA of fish EGFR for inter-paralog amino acid inversions using DIRphy. We decided to compare the Cypriniformes clade with the other teleosts because of the high coverage of genomes in both groups, and a sufficiently long separation between the two groups to allow functional divergence on the protein sequence (Fig 3A). We observed a distribution of the scores that resembles an extreme-value distribution, with most of them below 0.01 (Fig 3B). Using the theoretical model, we calculated that the expected value for the probability of inter-paralog inversions in the same tree is 0.002, much lower than the score of the 99th percentile of 0.16. This percentile value was used as a threshold to select eight sites, the majority of which (six) were from the EGFR extracellular domain. We highlighted the positions of the six sites on the 3D structure of EGFRa and EGFRb extracellular domain from a representative Cypriniformes (*S*. *anshuiensis*), modeled using the human structure template (Fig 3C). Of the six selected positions, only one (MSA pos 520) was found at the ligand-binding pocket interface. Pos 520 corresponds to Phe-357 in hEGFR. Previous studies showed that the hydrophobic interaction between Phe-357 and Tyr-13 in the ligand hEGF is determinant for the binding [46]. In the fish clade *Xiphophorus*, the observed change between Phe and His at this position is considered to be the determinant cause of the different responses of EGFRa and EGFRb after ligand stimulation [47]. Out of the six positions in the extra-cellular domain, two showed a conservative substitution (Fig 3D). MSA position 506 contains hydrophobic and aliphatic amino acids (Ile or Val), while position 491 shows a small and uncharged amino acid (Ser or Thr). All other positions exhibited a shift of amino acid physicochemical properties. These results show that DIRphy can identify inter-paralog amino acid inversions in a protein duplication phylogeny, regardless of the amino acid substitution type.

### Score validation by simulated evolution

**Fig 4:**
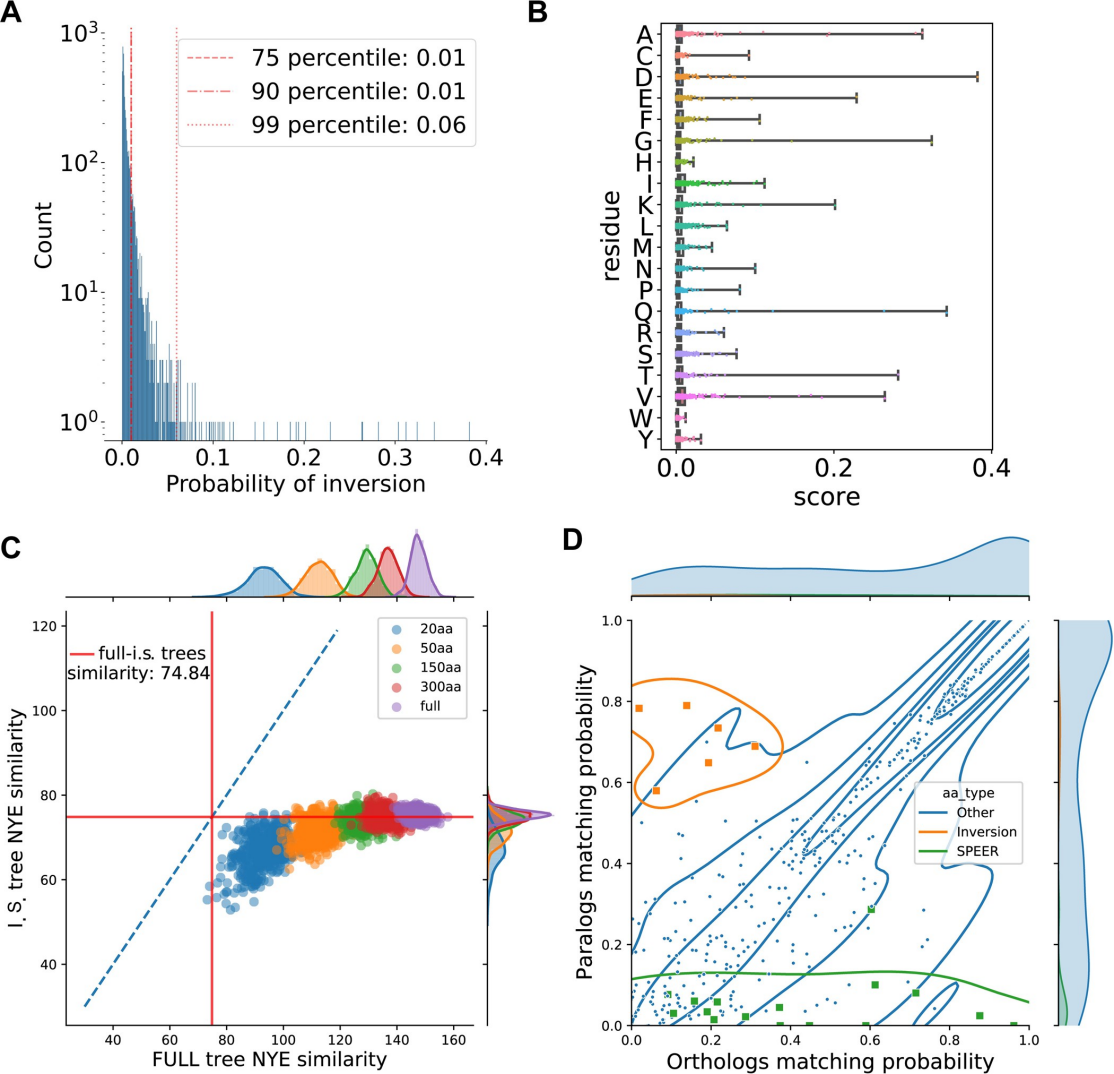
Outcome to the validations. **(A)** Distribution of the simulated evolution DIRphy scores. A 5000 random amino acid sequence was evolved through the fish EGFR phylogenetic tree using the same evolutionary models used to generate the tree. The resulting MSA was used to compute the DIRphy score. **(B)** Distribution per amino acid of the simulated evolution DIRphy scores as shown by the reference (*S*. *anshuiensis* EGFRa). **(C)** Bootstrap trees similarity to the full and inverted sites trees. The color represents the length of the sub-alignment used to generate the bootstrap tree. The red line shows the similarity between the full and inverted sites trees. The blue line is the identity line. **(D)** Comparison of the sites identified by DIRphy and SPEER. The matching probability of the HMM of four sub-alignments was used to compare between species the orthologs (EGFRa vs EGFRa, EGFRb vs EGFRb) and paralogs (EGFRa vs EGFRb). The matching probability is calculated as the average of two dot products of the frequency arrays. The orange color shows sites where an inversion was identified, while the green color shows sites where the p-value of SPEER score is lower than 0.01.

We statistically validated the score observed in the fish EGFR data using a simulated evolution experiment. In this simulation, random starting amino acids are run through a phylogenetic tree that has the same topology as the previously computed fish EGFR tree. The simulation used the same evolutionary model of the fish EGFR tree to output a MSA as a result. Compared to the fish EGFR MSA, the simulated evolution MSA showed on average lower DIRphy scores while having a similar shape of the score distribution (Fig 4A). No specific amino acid was found to have high scores. Interestingly, the three amino acids involved in the interaction in position 520 (His, Tyr, and, except for one site, Phe) failed to produce any score higher than 0.05 in the simulation (Fig 4B). For further analysis, we used the 99^th^ percentile score of the simulation as a threshold for selecting inverted residues in the real dataset (S5 Fig and S1 Table). In summary, the simulated evolution experiment provided a score threshold for detecting residue inversions and confirmed the low chance of this event in the fish EGFR dataset, as observed in the theoretical model.

### Tree bootstrap

We characterized the phylogenetic information carried by the inverted sites when reconstructing the correct fish EGFR phylogeny tree structure. First, we computed a phylogenetic tree using the sub-alignment of 19 inverted sites that score higher than the 99^th^ percentile in the simulated evolution experiment (S6 Fig). In this phylogenetic tree, we can still see two defined groups of sequences corresponding to the two copies of EGFR. Though, as expected, all the sequences from Cypriniformes EGFRa cluster together with the EGFRb group, and vice versa the Cypriniformes EGFRb cluster in the EGFRa group. When we compared this tree to the full-alignment tree, we calculated a similarity value of 74.84 out of 165 using the tree similarity score of Nye *et al*. [50]. Next, we generated a pool of bootstrap trees with a reduced alignment length, and we checked their similarity to the full alignment and the inverted sites trees (Fig 4C). We observed a decrease in tree similarity to the full tree proportional to the decrease in alignment length. However, the inverted sites tree distance is statistically less similar to the full alignment tree than the bootstraps, even among the bootstraps with an equivalent length of 20 amino acids (student t-test p-value: 0.0019). This result suggests that the inverted sites are just a minority of the sites in the alignment and that they disperse the phylogenetic information faster than the average. This view is compatible with the hypothesis that a few functional substitutions are hidden behind an overwhelming amount of neutral or nearly-neutral variants, complicating their detection.

### Comparison of DIRphy inversions to Specificity Determining Sites (SDS) prediction

We predicted the SDS of fish EGFR using SPEER [32], and observed a marked difference in the type of identified positions compared to DIRphy. To compare the two methods, we defined a “matching probability” as the dot product of the amino acid emission probability vectors of two HMM models. Then, we compared, for each site of the fish EGFR alignment, the mean matching probability between HMM models of the orthologs (cypriniformes EGFRa to other teleosts EGFRa, and cypriniformes EGFRb to other teleosts EGFRb) with the mean matching probability between the paralogs (cypriniformes EGFRa to other teleosts EGFRb, and cypriniformes EGFRb to other teleosts EGFRa) (Fig 4D). From this analysis, it is evident that SPEER tends to predict sites with a high ortholog-low paralog correlation. These sites are likely to possess a paralog-specific function. On the other hand, DIRphy identifies diametrically opposite sites, with a high paralog-low ortholog correlation. Both types of sites deviate from the diagonal, a pattern that suggests a functional adaptation. However, the sites that show an inversion are more challenging to identify for SDS predictions because, for a subset of species, the conservation pattern is inverted, and the signal is averaged out. In summary, the identification of inter-paralog inversion events has the potential to improve functional residue predictions, as DIRphy is able to identify functional sites that are overlooked by SDS prediction methods.

### Molecular dynamics of two fish EGFR duplicates bound to a cognate ligand

To explore a functional relationship of the identified residues, we performed molecular dynamics simulations of the fish EGFR-EGF complex. First, we generated alpha fold 2.0 models of the EGFR in complex with EGF for paralogs in *Oryzias latipes* (olat EGFRa and b) and *Sinocyclocheilus anshuiensis* (sans EGFRa and b). We performed 100 ns MD in four replicates for each of the four samples: olat EGFRa, olat EGFRb, sans EGFRa, sans EGFRb. We observed stable simulations that had one main RMSD peak at 0.25 nm and a small secondary peak at about 5 nm (S7 Fig). In all simulations, the ligand did not leave the binding pocket where it was modeled, suggesting that the mode of binding did not change compared to the human structure. However, we observed a higher average in the fluctuations of the ligand residues in the olat EGFRb:EGF and sans EGFRa:EGF simulations. Accordingly, the number of hydrogen bonds between ligand and receptor followed the same trend. During the simulation, olat EGFRa:EGF has on average ~5 more hydrogen bonds to the ligand compared to olat EGFRb:EGF, while the opposite is true for sans simulations. Next, we observed the behaviour of position 520 of the MSA, previously identified with a high inter-paralog inversion score and directly in contact with the ligand. We see that when this position is histidine, in olat EGFRb:EGF and sans EGFRa:EGF simulations, the average fluctuations are higher than the phenylalanine in the corresponding paralogs. To conclude, the MD results confirm the same trend we observe with the inter-paralog inversions at the functional level.

### Inter-paralog amino acid inversions in an extended protein dataset

**Fig 5:**
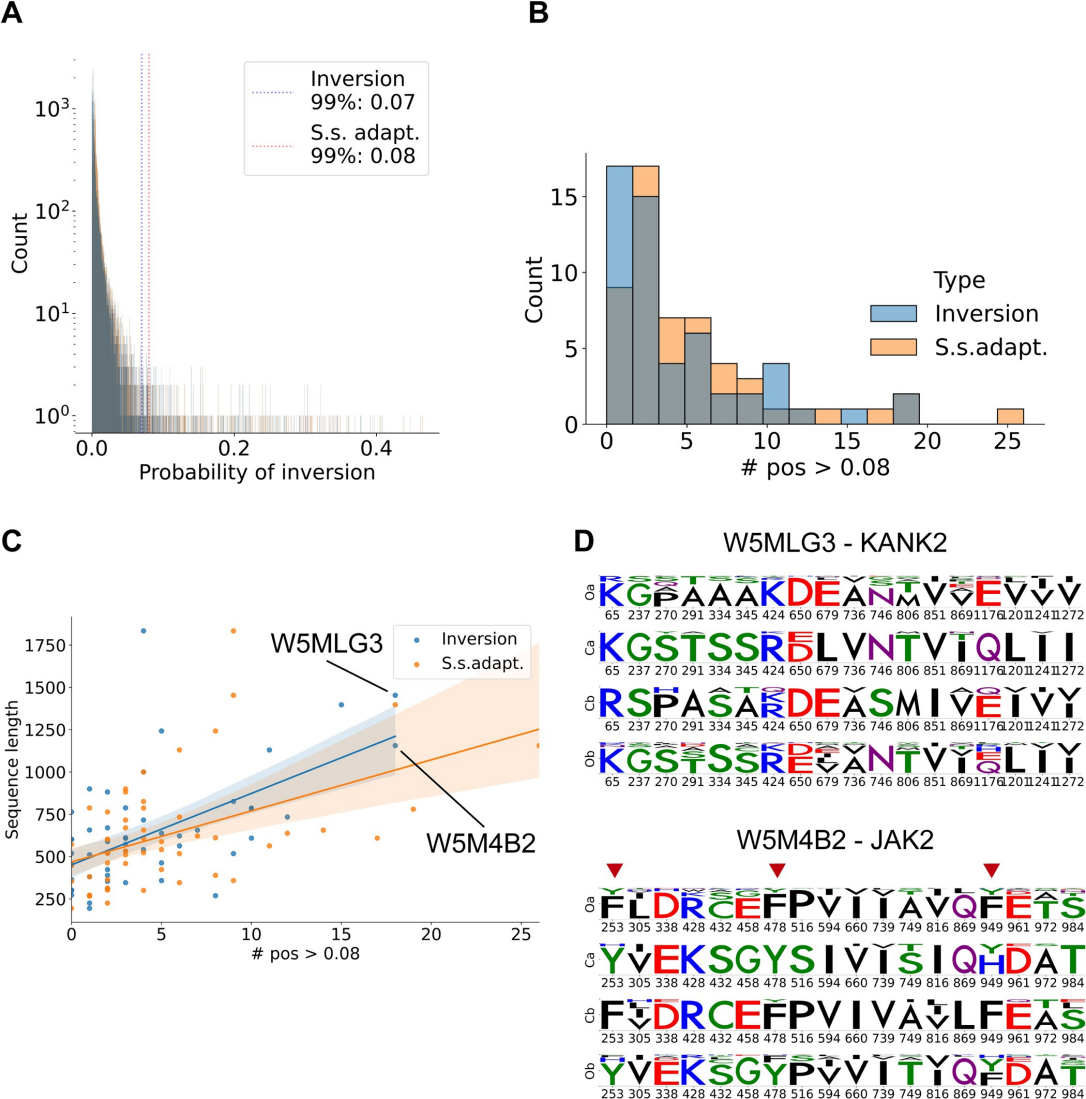
Extended protein dataset validation. **(A)** Distribution of DIRphy scores in the extended dataset. DIRphy was tested in a dataset of 54 protein duplication trees obtained from fish proteomes. The blue color indicates inter-paralog inversion scores, the orange color indicates specie-specific adaptation scores. The two lines show the 99^th^ percentile score of the distributions. **(B)** Number of positions with a score higher than 0.08 per protein. More than half of the dataset has less than 5 high scoring positions. **(C)** High scoring positions (>0.08) per protein versus protein length. The two proteins that have 18 high scoring positions in the inter-paralog inversion score are highlighted. **(D)** Sequence logo of the high scoring positions for the two previously highlighted proteins. The two names are respectively the spotted gar reference name and the human ortholog name. In red arrows, the positions that involve a tyrosine inversion are highlighted.

In order to obtain a wider view on inter-paralog inversion events, we tested our algorithm on an extended dataset of 54 duplicated protein trees found in teleost proteomes. The protein trees were selected among those having two proteins copies in at least 80% of Cypriniformes and 60% of other teleosts, and forming two separated clusters. As for the EGFR case, we searched inter-paralog inversion events and specie-specific adaptations between these two clades of fish. We observe again a distribution of scores with an extreme-value shape (Fig 5A). Comparing inter-paralog inversions events and specie-specific adaptations, we found a little difference between the background distributions. Therefore, the background distribution of both events provides the same reference score for the identification of outliers. The 99^th^ percentile of 0.07 shows that the number of inversion events resemble the one from the simulated evolution experiment rather than the EGFR example. In our reduced dataset, most proteins have less than 5 positions with a high score (Fig 5B). However, two proteins display an unusually high number of inter-paralog inversion events, similarly to the case of EGFR extracellular domain. While most proteins of similar length showed an average of 5 inversions, in EGFR extracellular domain we observed 19 inversions, while in spotted gar’s reference ID W5MLG3 and W5M4B2 we observed 18 inversions (Fig 5C). The two proteins respectively correspond to human orthologs KANK2 and JAK2. At least half of the positions do not appear to have a substantial modification of the amino acid physicochemical properties (e.g., a substitution to another hydrophobic amino acid). However, other sites show promising substitutions, like the leucine to glutamic acid in KANK2 ortholog position 679, and the three marked tyrosine sites in JAK2 (Fig 5D). The tyrosine sites are relevant because JAK2 is a tyrosin-kinase protein that is regulated by auto-phosphorylation and has several auto-phosphorylated tyrosine positions in its sequence. Interestingly, JAK2 is directly interacting with EGFR in human [51], showing that a broad analysis with our pipeline highlights the pathway potentially related to the specific function responsible of the inter-paralog inversions.

## Conclusions

In conclusion, we have observed an event in paralogs that lead to the inversion of functional residues. This new event that we named inter-paralog inversion has been described by a theoretical model and validated by literature and bioinformatics studies. Inter-paralog inversions are a distinct case of parallel evolution. While parallel amino acid replacements have been observed to be fixed at a rate comparable to the one of neutral replacements [52], we observe few inter-paralog inversions in the datasets we analyzed. Previously, an attempt to detect parallel and convergent evolution in large protein families with many duplications was already made by Von Der Dunk & Snel [53]. However, our study focuses on the distinct case (asymmetric divergence) where the diverging paralogs converge to the same phenotype. In such a case, neutral substitutions related to the ancestral relationship of a protein can be filtered out to better search for functional adaptations. To the best of our knowledge, we are the first to identify and describe the inter-paralog amino acid inversion event. These inversions are potentially exploited for functional divergence and, if missed, might lead to the wrong classification of proteins to functional groups. We provide a general tool, named DIRphy, to identify inter-paralog inversions in a large protein dataset. DIRphy can be easily integrated in an existing pipeline of protein annotation to improve functional annotation and provide the positions that might have been overlooked by other functional site prediction methods. On a large-scale analysis, DIRphy has the potential to provide insights into which pathway is affected by the inter-paralog amino acid inversions.

## Materials and methods

### Theoretical model

The model was built on the following assumptions: 1) equal branch length between the two paralogs: b1 = b2, b3 = b4 = b5 = b6; 2) only zero to one substitution can occur in each of the six branches; 3) after a substitution, each amino acid is equiprobable; 4) no selective pressure; 5) the probability of a substitution on a branch solely depends on the branch length (substitution rate) and is *P* = 1−*e^−λ^* where P is the probability of a substitution and *λ* is the substitution rate.

Given the probability of a substitution in each of the six branches, we can calculate the probability of all the 64 (2^6^) configurations of substitutions on the tree. A configuration unequivocally leads to a determined leaf node state (S1 Fig). We defined the leaf node states with the allowed values zero, one, or two. The state value represents the number of substitutions that happened in the branches connecting to the leaf node (Fig 1A). Then, we defined the probability of a match between leaf nodes based on the state value. The probabilities describe the situation in which an amino acid can change to one of 19 possible other amino acids. In some cases, the matching probability depends on the state of the ancestral node before speciation, e.g., a single or double mutation in the inner branch leaves. Finally, we defined seven categories based on the type of matching at the leaf nodes, and we defined their probability of occurrence based on the matching probabilities between leaf nodes.

### Model categories

Here we give a brief description of the model categories and formulas used to calculate their probabilities in the model.

*Conserved*

$$P_{cons}={mi}_{Xa,Ya}*{mi}_{Xb,Yb}*max({mo}_{Xa,Xb},{mo}_{Xa,Yb},{mo}_{Ya,Xb},{mo}_{Ya,Yb})$$

Where *mi* stands for the inner match, *mo* stands for the outer match and corresponds to the probabilities of a match in Fig 1B. The conserved category collects the states where a site is invariant in all four leaf nodes. It could arise from no mutations, but also from (two, three, or) four mutations to the same amino acid. In the formula, the maximum value of the outer matches is used as the best approximation of the conditional probability of matching all leaf nodes given the two inner matches.

*Type 1 Divergence*

$$\begin{array}{c} P_{type1}=\\ {=mi}_{Xa,Ya}*{(1-mi}_{Xb,Yb})*(1-min({mo}_{Xa,Xb},{mo}_{Ya,Xb}))*(1-min({mo}_{Xa,Yb},{mo}_{Ya,Yb}))\\ +{(1-mi}_{Xa,Ya})*{mi}_{Xb,Yb}*(1-min({mo}_{Xa,Xb},{mo}_{Xa,Yb}))*(1-min({mo}_{Ya,Xb},{mo}_{Ya,Yb})) \end{array}$$

The type 1 divergence collects the states where the amino acid is matching between species only in one paralog, while there is no match in the other paralog.

*Type 2 Divergence*

$$P_{type2}={mi}_{Xa,Ya}*{mi}_{Xb,Yb}*(1-P_{cons})$$

The type 2 divergence collects the states where the two paralogs display a different amino acid but are conserved between species. Type 1 and type 2 classifications are based on [54].

*Recent Divergence*

$$\begin{array}{c} P_{recent}=sum({mi}_{Xa,Ya}*{mo}_{Xa,Xb}*(1-{mo}_{Xb,Yb})\\ {mi}_{Xa,Ya}*{mo}_{Xa,Yb}*(1-{mo}_{Xb,Yb})\\ {mi}_{Xb,Yb}*{mo}_{Xa,Xb}*(1-{mo}_{Xa,Ya})\\ {mi}_{Xb,Yb}*{mo}_{Ya,Xb}*(1-{mo}_{Xa,Ya})) \end{array}$$

The recent divergence collects the states where only one leaf node is different (diverged) compared to the other three nodes.

*Inversion / Specie-Specific Adaptation*

$$P_{inv}={mo}_{Xa,Yb}*{mo}_{Xb,Ya}*(1-\min({mi}_{Xa,Ya},{mi}_{Xb,Yb}))$$

And similarly,

$$P_{ssa}={mo}_{Xa,Xb}*{mo}_{Ya,Yb}*(1-\min({mi}_{Xa,Ya},{mi}_{Xb,Yb}))$$

These two categories represent the states where the amino acid does not match between orthologs but matches between paralogs for the inversion or between species for the species-specific adaptation.

### Calculation of inter-paralog inversion score

We devised a score to identify inter-paralog inversions in a phylogeny. The score was based on the probability of observing an inversion in the previously described model and calculated with the following steps: 1) Divide an MSA into four sub-alignments (two EGFR copies and two species groups). 2) Generate four amino acid frequency arrays, optionally normalized by pseudo-counts*. 3) Calculate the probability of a match between two groups using the dot product of the frequency array. 4) Calculate the joint probability of inversion (or similarly for species-specific adaptation) from the conditional probabilities and the frequency array matching using the following formulas:

$$P_{\left(\bar{M(Xa,Xb,Ya,Yb)}|M(Xa,Yb),M(Ya,Xb\right)}=\sum_{i}^{20}\frac{Xa[i]*Yb[i]}{M(Xa,Yb)}*(1-\frac{Ya[i]*Xb[i]}{M(Ya,Xb)})$$

$$P_{\left(\bar{M(Xa,Xb,Ya,Yb)},M(Xa,Yb),M(Ya,Xb\right)}=P_{\left(\bar{M(Xa,Xb,Ya,Yb)}|M(Xa,Yb),M(Ya,Xb\right)}*M(Xa,Yb)*M(Ya,Xb)$$

Where *Xa*,*Xb*,*Ya*,*Yb* are the amino acid frequency arrays for the MSA sequence groups with the same names, *i* is the counter spanning each amino acid. The probability of a match (e.g., *M(Xa*,*Yb)*) is given by the array dot product: $M(Xa,Yb)=\sum_{i}^{20}Xa[i]*Yb[i]$. The latter joined probability represents the inter-paralog inversion score.

### * Pseudo-count normalization

To correct any possible bias given by groups with a small number of species, we implemented a pseudo-count normalization of the amino acid frequency array, as previously done in Tatsuov *et al*. [55]. We used the LG protein substitution matrix [56] as background amino acid frequency probability. The value of the beta parameter for the pseudo-counts formula was set by default to five; however, it is possible to modify this parameter before running the pipeline.

### Fish dataset

To test the inter-paralog inversion score, we generated a fish genome dataset. First, we downloaded all genomes from the European Nucleotide Archive (ENA) belonging to taxon 41665 (Actinopterygii). Through this method, we obtained 88 fish genomes. Next, we downloaded the pre-annotated fish EGFR protein sequences from the ENSEMBL database [57] and used them to build an HMM profile with the HMMER package [58]. The HMM profile was used as a query in Augustus package suite [59] to search for EGFR related genes in the fish genomes. We then filtered out interrupted CDS, sequences clustering with other ErbBs in a phylogenetic tree, and sequences with an aberrant branch length in the non-synonymous codon tree. After this procedure, we obtained 167 fish EGFR protein sequences.

### Phylogenetic analysis

We performed the phylogenetic analysis of the fish EGFR protein sequences using MAFFT [60] to align the sequences, and IQTREE [61] with ModelFinder [62] to search for the best evolutionary model to generate the phylogenetic tree. To generate the synonymous tree, we used paml CODEML [63].

### Sequence and structure analysis

The sequences and alignments were handled using Unipro Ugene [64]. The protein structure images and analyses were performed with UCSF Chimera [65]. The modelling of fish EGFR structures was performed using the SWISS-MODEL web server [66] and AlphaFold 2 [67].

### Simulated evolution

We ran a simulated evolution experiment using an in-house pipeline based on the Pyvolve python package [68]. The pipeline simulated an evolutionary pathway of 5000 random amino acids on the fish EGFR phylogenetic tree, using the same model of evolution and evolutionary rates that were used to construct the tree (JTT with rate heterogeneity) [69–71]. The simulation generated an output alignment that was used to run the DIRphy pipeline, to calculate the base probability of an inter-paralog inversion.

### Bootstrap

To perform the bootstrap, we used an in-house Matlab script. We selected from the fish EGFR DIRphy prediction the 19 sites with a score higher than the 99^th^ percentile of the simulated evolution scores. We calculated a phylogenetic tree for the full alignment and the inverted sites alignment using the neighbor-joining algorithm [72] and the BLOSUM80 matrix [73]. The similarity distance between trees was calculated using the method described in Nye *et al*. [50]. We then performed 500 bootstrap alignments for each set of alignment lengths: full, 250, 100, 20. We excluded columns with 90% or more gaps and repeated the sampling whenever one sequence did not have at least one non-gap position. For each bootstrap alignment, we generated a tree, then calculated the distance to the full tree and inverted sites tree.

### Molecular dynamics

We generated the models for the molecular dynamics simulations using alpha fold 2.0 multimer [74]. The sequence of *Oryzias latipes* (olat) and *Sinocyclochelius anshuiensis* (sans) EGFRa and EGFRb extracellular domains were extracted from the MSA used in the previous analyses. The sequences for the EGF ligands were extracted from the ENSEMBL database and confirmed using manual alignments and the sequences reported in Laisney *et al*. [41]. The models were cut of the disordered regions (pLDDT score < 50) at the N- and C- terminal of the proteins. We performed the molecular dynamic simulations using the same parameters and scripts as in our previous paper [75]. In short, we used Gromacs 2020.1 [76] and charmm36-mar2019 force field [77]. We solvated and neutralized the system with NaCl atoms in a dodecahedral box, then we performed energy, temperature, and pressure equilibrations. We ran a 100 ns production simulation in quadruplicates using the Verlet cut-off scheme for non-bonded interactions [78], Particle Mesh Ewald for long range electrostatic interactions [79], and the LINCS constraint algorithm [80]. The analysis of the trajectories was performed using the gromacs standard package and python scripts.

### Extended dataset

The extended dataset was obtained from 70 reference fish proteomes in UNIPROT database [81]. We used BLAST [82] to find all hits in each proteome using as query the duplication outgroup (*Lepisosteus oculatus*) proteome. The top 5 hits from each proteome were used to generate a MSA with MAFFT and a phylogenetic tree with IQTREE. Then, we selected 54 trees in which a clear and even duplication event with at least 80% of Cypriniformes and 60% of other Actinopterygii were present in two copies and evenly split in two groups. This dataset was then used in the subsequent analysis.

## Supporting information

S1 Fig Probability of a configuration. The state of a branch (zero or one) represents whether a mutation happened in that branch. The probability of the branch state solely depends on the corresponding branch length (rate of mutation): t1 for b1 and b2, t2 for b3 to b6. The product of the six branch states gives the probability of a tree configuration. From the six branch states, it is possible to reconstruct univocally the leaf node states by counting the number of mutations in the two branches connected to a leaf node.
(TIFF)

S2 Fig Fish EGFR phylogeny. Phylogenetic tree of the 167 EGFR proteins found in the fish genomes dataset. The label shows, in order, the name of the species where this EGFR was found, the contig name, start position, end position, the length in DNA bases, and the AUGUSTUS fastBlockSearch score, separated by underscores. The coloring shows how many genes are found in the species of this EGFR. The annotated sequences of zebrafish and tilapia EGFR were taken from the ENSEMBL database and added to the tree for reference. The main nodes bootstrap values are shown.
(TIFF)

S3 Fig Synteny comparison of EGFRa and EGFRb in zebrafish and medaka. The ENSEMBL genome browser was used to perform a synteny analysis of the loci containing EGFRa and EGFRb in zebrafish and medaka fish. It appears that the regions of the two genes corresponds at the chromosome level.
(TIFF)

S4 Fig Synteny comparison of EGFRa and EGFRb in zebrafish and tilapia. The ENSEMBL genome browser was used to perform a synteny analysis of the loci containing EGFRa and EGFRb in zebrafish and tilapia fish. It appears that the regions of the two genes corresponds at the chromosome level.
(TIFF)

S5 Fig Logo of the fish EGFR high DIRphy scoring inverted residues. The output of DIRphy for the sites of the phylogeny of fish EGFR in which the score is higher than the 99% percentile of the simulated evolution experiment (>0.07). Oa and Ob stands for Other fish EGFRa and EGFRb, while Ca and Cb stands for Cypriniformes EGFRa and EGFRb.
(TIFF)

S6 Fig Comparison of the inverted residues tree. The two trees made from the full alignment (left) or the inverted residues sub-alignment (right) are colored by the four groupings used to calculate the DIRphy score: Cypriniformes EGFRa (red), other fish EGFRa (orange), Cypriniformes EGFRb (blue), other fish EGFRb (teal).
(TIFF)

S7 Fig Molecular dynamics of two fish EGFR duplicates in complex with the cognate ligand. Four simulations of 100 ns of the alpha fold model of EGFRa and EGFRb in complex with EGF were performed for *Oryzias latipes* (olat) and *Sinocyclocheilus anshuiensis* (sans) proteins. **(A)** the root mean square deviation (RMSD) of the trajectories. The peak at ~0.25 nm is high in all four types of simulations. However, a secondary peak is present at ~5 nm, slightly higher in olat EGFRa and sans EGFRb. **(B)** The EGF ligand root mean square fluctuation (RMSF) over the course of the simulation. The line shows the average of the repeats. On average olat EGFRb ligands fluctuates more than in EGFRa, while the opposite is true for sans EGFRs. **(C)** The number of H-bonds between the ligand and the receptor, calculated using gromacs command *gmx hbond* using default parameters. On average, there are less H-bonds with the ligand in olat EGFRb and sans EGFRa. **(D)** The receptor RMSF on position 520 of the MSA, previously detected as inverted using the DIRphy pipeline. Interestingly, the histidine residues in olat EGFRb and sans EGFRa have a higher fluctuation on average than the corresponding phenylalanine residues in the other copy.
(TIFF)

S1 Table Fish EGFR high DIRphy scoring inverted residues. The output of DIRphy for the sites of the phylogeny of fish EGFR in which the score is higher than the 99% percentile of the simulated evolution experiment (>0.07). The grouping of species was set to be Cypriniformes vs all other fish. “Pos” shows the residue in the reference EGFRa, “Other pos” shows the residue in the reference EGFRb. “Conservation” shows the site conservation (identity) for all EGFRa or EGFRb in the MSA jointly. The reference species for this analysis was set to *S anshuiensis*.
(XLSX)

S2 Table Comparison of EGFRa and EGFRb from a representative each for the Cypriniformes and other teleosts clades. The EGFRa and EGFRb gene annotations was collected in ENSEMBL genome browser for the two copies in *Clupea harengus* (herring, cypriniformes) and *Oryzias latipes* (medaka). The table shows the respective sequence identity of the genes alignments, the Gene Order Conservation (GOC) score, and the Whole Genome Alignment (WGA) score. The GOC score represents how many of the shared neighboring genes are preserved with the same order, while the WGA score is a measure of the similarity that considers parts of the non-coding sequence. A full description of how the scores are calculated can be found on the ENSEMBL website.
(XLSX)
