# Supplementary figures and images for: Inter-paralog amino acid inversion events in large phylogenies of duplicated proteins
Source: PLoS Comput Biol. 2022 Apr 4;18(4):e1010016. doi: 10.1371/journal.pcbi.1010016 (PMC9009777; doi:10.1371/journal.pcbi.1010016)

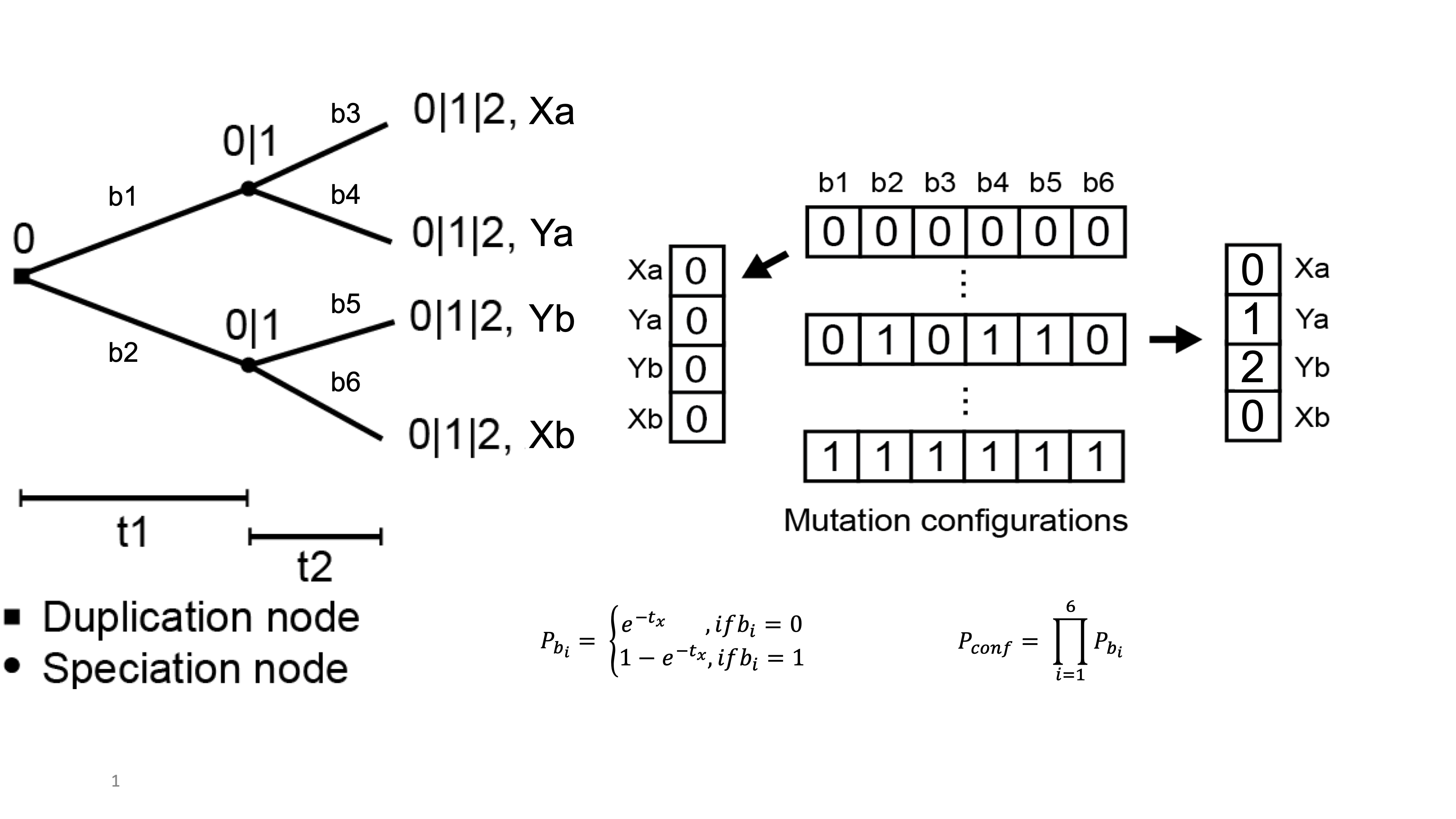

Supplement: S1 Fig — The state of a branch (zero or one) represents whether a mutation happened in that branch. The probability of the branch state solely depends on the corresponding branch length (rate of mutation): t1 for b1 and b2, t2 for b3 to b6. The product of the six branch states gives the probability of a tree configuration. From the six branch states, it is possible to reconstruct univocally the leaf node states by counting the number of mutations in the two branches connected to a leaf node. (TIFF) [file pcbi.1010016.s001.tiff]

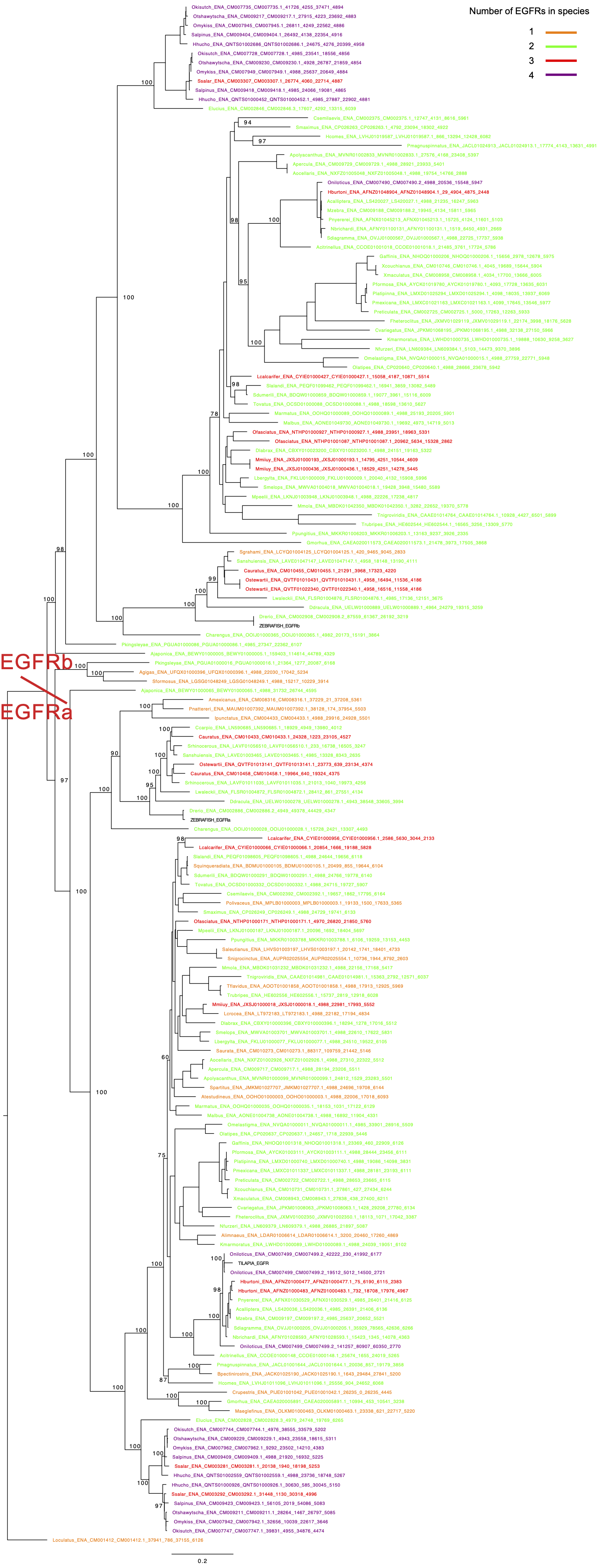

Supplement: S2 Fig — Phylogenetic tree of the 167 EGFR proteins found in the fish genomes dataset. The label shows, in order, the name of the species where this EGFR was found, the contig name, start position, end position, the length in DNA bases, and the AUGUSTUS fastBlockSearch score, separated by underscores. The coloring shows how many genes are found in the species of this EGFR. The annotated sequences of zebrafish and tilapia EGFR were taken from the ENSEMBL database and added to the tree for reference. The main nodes bootstrap values are shown. (TIFF) [file pcbi.1010016.s002.tiff]

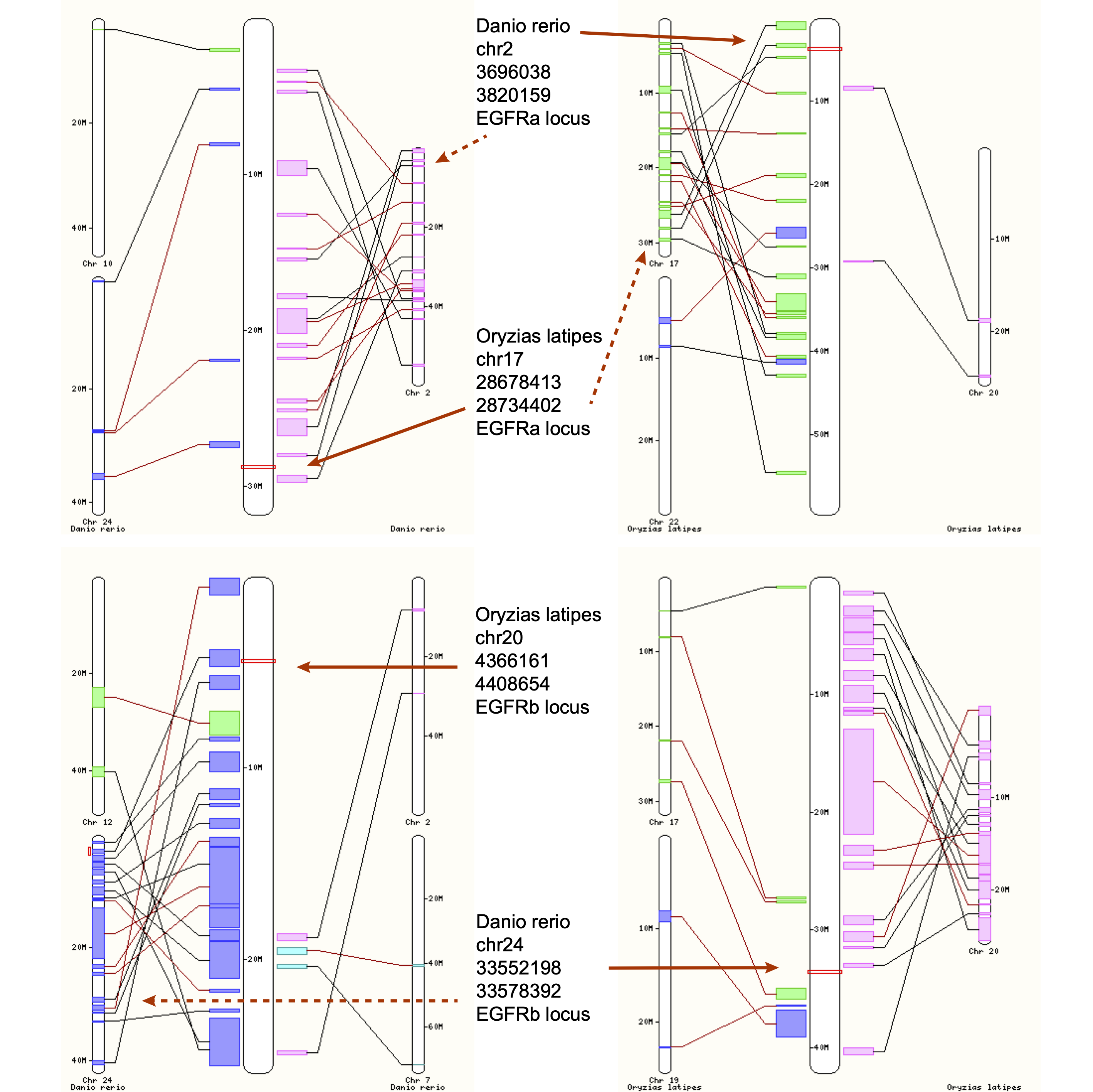

Supplement: S3 Fig — The ENSEMBL genome browser was used to perform a synteny analysis of the loci containing EGFRa and EGFRb in zebrafish and medaka fish. It appears that the regions of the two genes corresponds at the chromosome level. (TIFF) [file pcbi.1010016.s003.tiff]

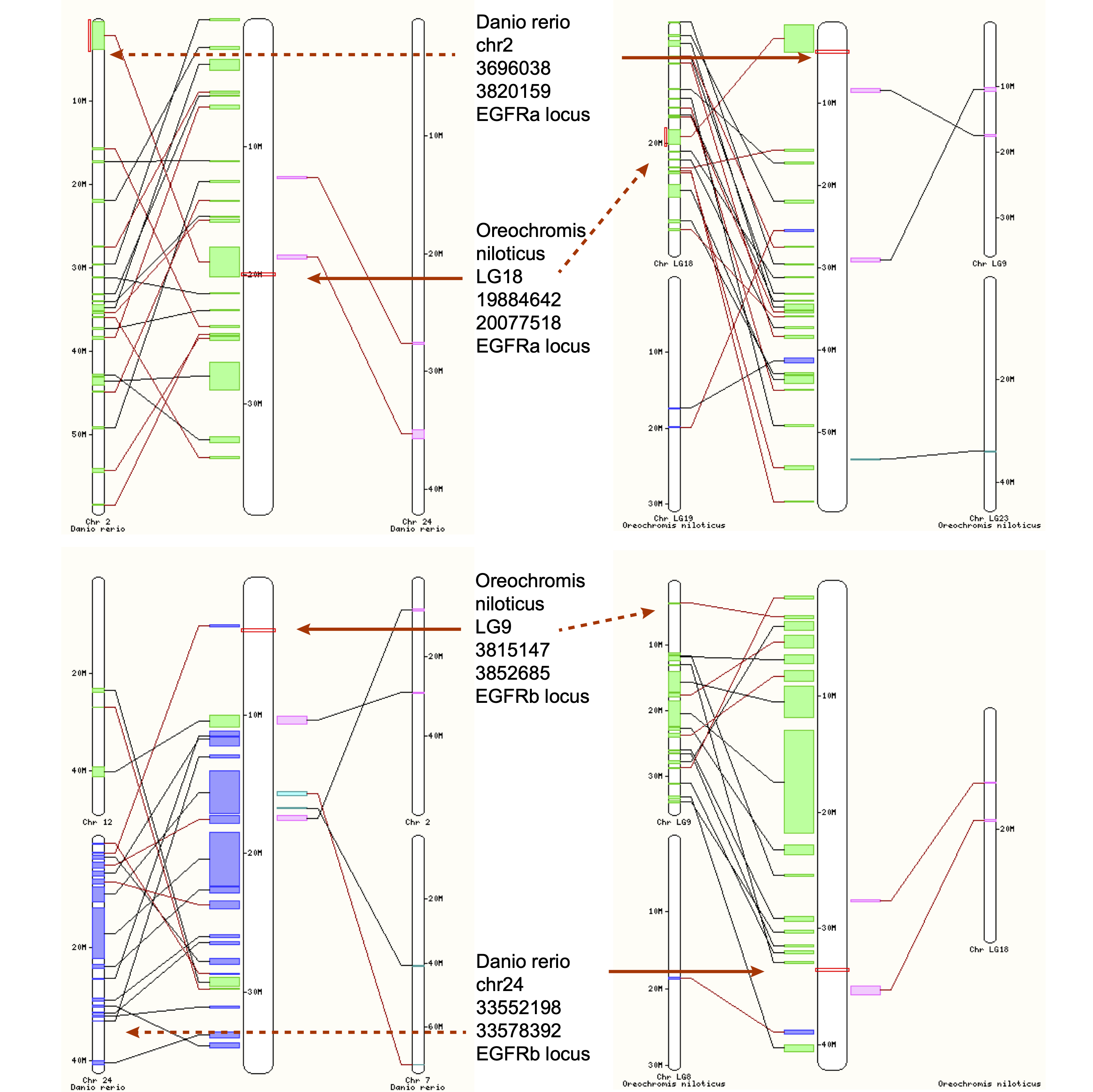

Supplement: S4 Fig — The ENSEMBL genome browser was used to perform a synteny analysis of the loci containing EGFRa and EGFRb in zebrafish and tilapia fish. It appears that the regions of the two genes corresponds at the chromosome level. (TIFF) [file pcbi.1010016.s004.tiff]

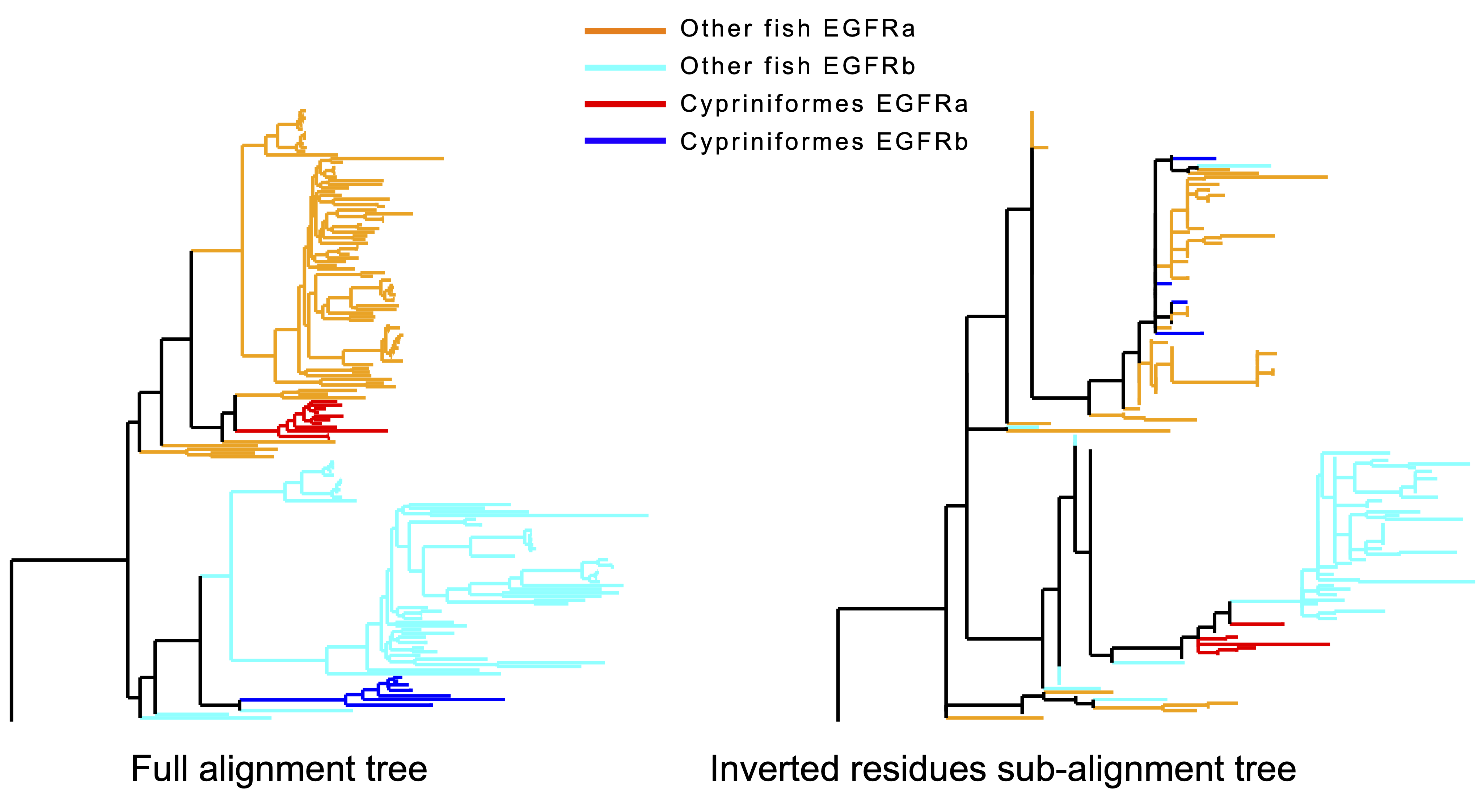

Supplement: S6 Fig — The two trees made from the full alignment (left) or the inverted residues sub-alignment (right) are colored by the four groupings used to calculate the DIRphy score: Cypriniformes EGFRa (red), other fish EGFRa (orange), Cypriniformes EGFRb (blue), other fish EGFRb (teal). (TIFF) [file pcbi.1010016.s006.tiff]

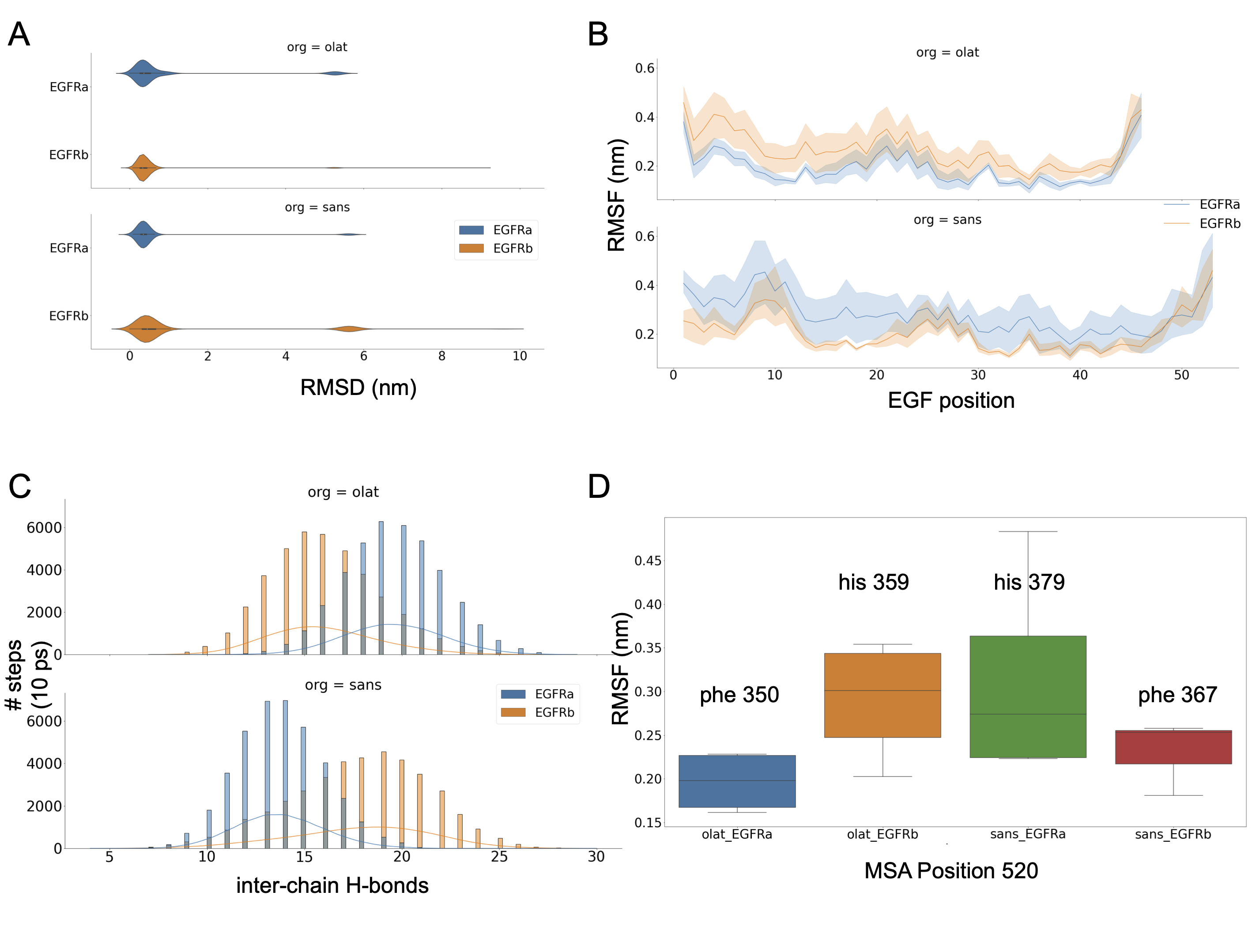

Supplement: S7 Fig — Four simulations of 100 ns of the alpha fold model of EGFRa and EGFRb in complex with EGF were performed for Oryzias latipes (olat) and Sinocyclocheilus anshuiensis (sans) proteins. (A) the root mean square deviation (RMSD) of the trajectories. The peak at ~0.25 nm is high in all four types of simulations. However, a secondary peak is present at ~5 nm, slightly higher in olat EGFRa and sans EGFRb. (B) The EGF ligand root mean square fluctuation (RMSF) over the course of the simulation. The line shows the average of the repeats. On average olat EGFRb ligands fluctuates more than in EGFRa, while the opposite is true for sans EGFRs. (C) The number of H-bonds between the ligand and the receptor, calculated using gromacs command gmx hbond using default parameters. On average, there are less H-bonds with the ligand in olat EGFRb and sans EGFRa. (D) The receptor RMSF on position 520 of the MSA, previously detected as inverted using the DIRphy pipeline. Interestingly, the histidine residues in olat EGFRb and sans EGFRa have a higher fluctuation on average than the corresponding phenylalanine residues in the other copy. (TIFF) [file pcbi.1010016.s007.tiff]
