# Supplementary material for: Inter-paralog amino acid inversion events in large phylogenies of duplicated proteins
Source: PLoS Comput Biol. 2022 Apr 4;18(4):e1010016. doi: 10.1371/journal.pcbi.1010016 (PMC9009777; doi:10.1371/journal.pcbi.1010016)
Supplement: Attachment — Submitted filename: 122013_1_rebuttal_2297993_r7nsrx.docx [file pcbi.1010016.s010.docx]

Dear Dr. Sara Monaco,

A point-by-point response (in black text) to each of the editorial and reviewer comments (in grey text) is presented herein. We would like to thank reviewers for their comments, which we consider to have been insightful, constructive and helpful. We think the manuscript considerably improved. A version of the manuscript with changes highlighted in yellow is enclosed as well as a “clean version” without any highlighted part.

Thank you very much in advance.

Yours sincerely,

Paola Laurino

------------------------------------------------------------------------------ 
Reviewer #1 (Evidence, reproducibility and clarity (Required)): 

This article focuses on one possible outcome of protein sequence evolution after duplication, in which the residue distribution at specific positions of a multiple sequence alignment becomes uncoupled from the distribution expected from the phylogeny of the protein family. The authors call these events "residue inversions" and interpret them as the result of functional pressures on family members with diverging cellular roles. Based on a theoretical model of residue evolution after duplication of the coding gene, the authors describe the criteria for categorizing a particular position in a protein as a "residue inversion" and develop an algorithm to identify such events in a multiple alignment. They then apply their approach to the family of Epidermal Growth Factor Receptors in Teleost fishes and identify 19 EGFR positions in a dataset of 88 fish genomes, which satisfy the criteria of "residues inversions". They provide support to the scoring scheme used in their approach through a simulated evolution run and conclude from a comparison of their positions to the ones predicted by SPEER to represent Specificity Determining Sites that the two are largely orthogonal and may therefore complement each other in sequence-based function prediction. 

Major comments: 
1. Throughout the paper, the functional involvement of positions subject to "residue inversions" is indirect, inferred from the literature, and in parts sparse and tenuous. It therefore remains unclear to what extent the interpretation that "residue inversions" represent functional adaptations is correct. The authors acknowledge this uncertainty in several places, including the Conclusions.

We agree with the reviewer that without experimental validation an uncertainty about the data interpretation remains, however testing protein function on a large scale and in non-model organisms is extremely challenging. Since we were aware of this obstacle, we validate our conclusions in different ways: 1. the theoretical model and the simulated MSA both show a lower chance of observing residue inversions than what we detected in the teleost fish EGFR example. 2. previous literature highlighted an identified inverted residue as the possible cause of sub-functionalization of teleost fish EGFR. 3 We generated the alpha fold models of teleost fish EGFR and performed molecular dynamic simulation of the two copies, in complex with the ligand. In our simulations, we see the same trend that we observe with the inter-paralog inversions at the functional level. The new results have been integrated in line 692-706.

2. "Residue inversion" is a very unintuitive term, which took me several readings to penetrate and made reading the article difficult. The authors may wish to reconsider this term. Naively, a residue inversion would be the swapping of residues between two positions, such that a residue expected in position A is found in position B, while the residue expected in B is found in A. That is what I suspect most readers will think. 

We acknowledged that the terminology might be confusing. We therefore decided to define it as inter-paralog inversion of amino acids throughout all the text.

3. Is the phenomenon described here just a curiosity, or an important aspect of divergent evolution after duplication? The authors seem to be of two minds about it, calling the phenomenon "rare" in the Abstract, but an "important and understudied outcome of gene duplication" in the Introduction, then hedging again that it "might be rare" in the Conclusions. The benefits of recognizing such positions are also formulated with great caution, for example in lines 309-311: "In summary, the identification of residue inversion event has the potential to improve functional residue predictions". 

We agree with the reviewer that we did not yet test the recurrence of this event on a large scale, however this does not exclude that this event is frequent. This work is focused on the observation, characterization, and implications of this event. Considering this comment and the one below we decided to perform a further analysis (see below for more details).

Additionally, the analysis of the frequency of this event at the whole-organism scale on multiple organisms, while interesting, would be out of the scope of this paper, if not just because it requires a totally different (large-scale) approach compared to the one used in here. This type of analysis is also limited by the absence of a database collecting intermediate knowledge that would speed up the initial part of ortholog classification at a broad range.

Finally, by rarity we mean the statistical chance of the event, not considering the effective chance of observing it from the real data. In fact, we rectified in the text using the reviewer’s observation.

OLD VERSION (ppXX):

Our work uncovers a rare event of protein divergence that has direct implications in protein functional annotation and sequence evolution as a whole.

NEW VERSION:

Our analysis shows a new way to investigate an important and understudied outcome of gene duplication.

It would probably strengthen the article substantially if the authors would (I) use their program to scan a large number of multiple alignments in order to establish more reliably how frequent this phenomenon actually is, and whether it is universal or a specifc aspect of eukaryotic, maybe even only vertebrate evolution; and then (II) mapped the positions identified on structural models for the proteins, obtained by homology modeling or AlfaFold prediction, in order to substantiate their potential origin as functional adaptations. 

We thank the reviewer for the thoughtful suggestions. (I) we tested the inter-paralog inversion score at the proteome level using a reduced dataset (70) of reference teleost fish proteomes from Uniprot. We obtained 54 proteins that duplicated in the teleost specific whole genome duplication, then we run our pipeline on it. We found that the overall distribution of scores is more similar to the simulated evolution experiment rather than to the EGFR test case. We integrated the new results and discussion in a new paragraph and new figure in line 708-716.

(II) We considered also the analysis requested in the second point. Unfortunately, we could not extract any meaningful data from the AlphaFold models.

Reviewer #1 (Significance (Required)): 

A method to improve the functional annotation of proteins in a paralogous family would be very useful, given the abundance of sequence data.

We thank the reviewer for acknowledging the importance of the question that we have addressed.

I am knowledgeable in varios aspects of molecular evolution and functional annotation. I am neither a mathematician, nor a developer of phylogenetic methods, so I cannot judge these aspects of the paper. 


------------------------------------------------------------------------------ 
Reviewer #2 (Evidence, reproducibility and clarity (Required)): 

Review of Pascarelli and Laurino titled “Identification of residue inversions in large phylogenies of duplicated proteins” 

I find the topic of the paper very exciting and long overdue. Indeed, I was under the impression that the question of parallel evolution in paralogous copies must have been addressed long ago: to my surprise, having looked in depth at the literature, that is only partially so. The manuscript, therefore, addresses a relatively novel and fundamental question of broad interest.

☺ 

Having said this, I also found the manuscript to suffer from an identity problem, which in many places encroaches on the underlying quality of the science. I will structure my review into three concerns: the identity issues, the novelty issue and the emergent quality issues from the two.

Identity issues: 

The manuscript is primarily dealing with an evolutionary issue – or I am biased to see it this way as an evolutionary researcher myself. Nevertheless, much of the language and terminology of the paper either misuses evolutionary terms or invents new ones in its place with a bias towards a protein chemistry perspective. Specifically, what the authors call “residue inversions” is called “parallel evolution” or “convergent evolution” in the literature. Also, "residues" are typically used for physical amino acids in a structure. If we are talking about sequence level “amon acid” would be a better term. The issue is further confounded by the meaning of “inversion” in genetics as a single mutation that inverts the position of nucleotides (i.e. an “AT” becomes “TA”). 

I strongly recommend for the authors to become familiarized with the common usage of existing and widely used terms in evolutionary biology that describe the phylogenetic patterns they see: parallel evolution, convergent evolution, homoplasy, etc, and to use them consistently throughout the manuscript. 

The same goes for "mutation", which the authors confuse on two levels: evolutionary and biochemical. Sometimes the authors refer to “mutation” of amino acids (which can be entertained at some level, but from a genetic perspective only nucleotides mutate – in the protein biochemistry field this term is frequently applied to amino acid residues, which is the basis of the identity issue). However, since the authors also use “mutation” to refer to a “substitution” (which is what we call a mutation that has become fixed in evolution) this creates another level of confusion. I urge the authors to change this aspect of the language of the manuscript to better reflect evolutionary concepts. 

As part of the language issues I am not sure how meta-functionalization in the author’s view differs either from neofunctionalization or specialization of duplicated genes. 

We thank the reviewer to point out the terminology issue, this will also help reaching a broader audience. We clarify the confusion surrounding the terms “mutation” and “residue inversion” by changing the former to “substitution”, while the latter to “inter-paralog inversions” (see also other reviewer comments).

We understand the importance of the usage of the correct term to talk about this event of protein sequences evolution. Therefore, we used convergent and parallel evolution accordingly when we discussed the nuances between Metafunctionalization and parallel evolution in the text, in lines 188 and 399.

Novelty issues: 

As I mentioned, the issue of parallel evolution of gene duplications is an extremely interesting topic. I was sure that the people who studied parallel evolution, or those interested in gene duplications, must have published extensively on this. However, my search of the literature revealed only a modest pre-existing effort. Nevertheless, previous efforts are not entirely non-existent and should be cited and discussed in this paper too. The most pertinent example is 

<https://bmcecolevol.biomedcentral.com/articles/10.1186/s12862-020-01660-1> 

which has an identical setup from what I can tell (compare Figure 1 in each paper). 

This paper was not hard to find using "parallel evolution", thus my focus on the language issues in the previous section. 

We thank the reviewer for his suggestion, we included the relevant papers in the text in lines 520-523. Interestingly, the cited paper shows that a comprehensive analysis of the fate of duplicated genes at the sequence level was done. However, in this paper, the ‘fate’ of a paralog is determined by counting the number of sites that support one or the other fate, independently of the orthologous relationship. In our study, we start from the orthologous relationship to pre-determine the fate of the paralogous protein, then we identify the sites that break this assumption. Our type of analysis is deemed to work only where the orthologous relationship is unequivocal. That is the reason why we chose an example with relatively short branch lengths after duplication (the teleost specific duplication). Our rationale is that with a higher genome coverage across organisms, resolving the orthologous relationship will get easier in time. However, our study focuses on a distinct case (asymmetric divergence) where the diverging paralogs converge to the same phenotype. In such a case, neutral substitutions related to the ancestral relationship of a protein can be filtered out to better search for functional adaptations.

Content issues: 

The lack of attention to evolutionary concepts, in my opinion, provided some missed opportunities for the authors to attack the problem in a more convincing fashion. Specifically, in the setup to distinguish between parallel evolution of paralogues versus orthologues ("inversion" versus "species-specific adaptation" in the author's text) one must be able to distinguish between the two copies and assign true evolutionary relationship. In practice, that is not always possible based on tree lengths or topologies alone because of confounding factors such as independent duplications or gene conversion events. 

I would feel better about the results of this study if the following two things were integrated. 

The use of synteny to better determine homologous relationships (declare copies to be true paralogues if they occupy the same syntenic region). 
To compare the frequency or parallel evolution of paralogues versus orthologues as a null model of the expected number of parallel events in paralogous copies.

We agree that a synteny analysis has to be included. We tested it for the EGFR proteins in fish and the results support the orthologous relationship of EGFRa and EGFRb in the two groups compared (Cypriniformes versus other teleosts). The results were included in the text and in the Supplementary figure in lines 303-305.

The second point targets the way the model derives the expectations: at the author's own admission the model makes a number of unrealistic assumptions, ") equal branch length between the two paralogs; 2) only zero to one mutation can occur in each of the six branches; 3) after a mutation, each residue is equiprobable; 4) no selective pressure; 5) the probability of a mutation on a branch solely depends on the branch length (mutation rate). The authors do not really test the resulting tree on deviation from these assumptions (I am sure that it does not conform) but essentially comparing the occurrence of parallel events in paralogues versus orthologues may solve the problem with a less restrictive set of assumptions (that one expects an equal number of parallel events in paralogues and orthologues unless there is some paralogue-specific selection pressure, which is what the authors are looking for. 

We compared the occurrence of the two outcomes in both the simulation and in the real data. In all cases, the two score distributions have a very similar shape, with a 99^th^ percentile score of respectively 0.062 and 0.113. Most sites in an alignment (>99%) are not expected to be inverted and will have scores very close to 0, making the identification of inversions a quest for outliers. Furthermore, in case of the real data, each distribution can be independently affected by different selective pressures that might bias the background distribution. While the inversion in paralogs is expectedly involving few, functional, residues, the inversion in orthologs is expected to have a broad effect. For example, a temperature adaptation might shift the number of polar residues on the protein surface (see for example: <https://academic.oup.com/peds/article/13/3/179/1466666>). Also, a different protein chosen for analysis might generate a different background distribution of the two events. In the larger dataset, the similarity of the two distributions is even more (99^th^ percentile of 0.07 and 0.08). Because of the shown similarity of the two event distributions, and the possible issues with different selective pressures, we leave the analysis suggested by the reviewer as a post-processing possibly performed by the user. We report a summary of this result born from the reviewer’s observation in line 478.

In summary, I believe that the topic is very interesting, the authors potentially found a new aspect of evolution of a specific gene family. However, in my opinion a major revision is needed to unite this text with the terms in the field, the previous publication and to integrate the two additional analyses I suggested. 


Minor Comments: 

I started adding these specific comments before generalizing the broader deviation from the common evolutionary language. There are more further along in the manuscript, but in the interest of time I will not articulate them here hoping that the authors will first try a major revision targeting these issues. 

Line 64: While neutral mutations help to determine the phylogenetic position of a protein, mutations of functional residues are a signal of functional shifts that might occur independently of the phylogeny. - this is quite misleading. All substitutions (neutral or beneficial) have a phylogenetic signal. In any case, this is discussed here in phylogenetic terms: <https://pubmed.ncbi.nlm.nih.gov/10742039>/ 

We corrected the sentence to refer to divergence time instead of phylogenetic signal.

OLD VERSION:

While neutral mutations help to determine the phylogenetic position of a protein, mutations of functional residues are a signal of functional shifts that might occur independently of the phylogeny.

NEW VERSION:

While neutral substitutions are directly proportional to the time of divergence, a change in functional residues could be a signal of a functional shift that might occur independently of the divergence time.

Line 107: "under high evolutionary pressure" - I do not know what evolutionary pressure is nor why it can be high or low. 

We corrected the term to “selective pressure”.

OLD VERSION:

Lorin et al. showed that both copies of EGFR might have been retained because they are involved in the complex process of skin pigmentation (40), which is under high evolutionary pressure in most fish.

NEW VERSION:

Lorin et al. showed that both copies of EGFR might have been retained because they are involved in the complex process of skin pigmentation (40), a trait that is under selective pressure in most fish

Line 112 "linearly inherited across orthologs" - linear is a poor choice of a word here. The first thing that comes to my mind is quadratic inheritance as an alternative. Perhaps the authors are looking for "vertical" versus "horizontal" - these are established terms in phylogenetics (think "horizontal gene transfer"). 

We corrected the term to “vertically inherited”.

OLD VERSION

Therefore, the power to predict functional residues is limited by our ability to track protein function on the phylogenetic tree when it is not linearly inherited by orthologs.

NEW VERSION

Therefore, the power to predict functional residues is limited by our ability to track protein function on the phylogenetic tree when it is not vertically inherited by orthologs.
It is my invariant practice to reveal my identity to the authors, 

Fyodor Kondrashov 


Reviewer #2 (Significance (Required)): 

Addressed in the above
